# Identification of the Prognostic Factors for Synchronous Multiple Primary Lung Cancer Treated With Staged Bilateral Surgery

**DOI:** 10.1111/crj.70017

**Published:** 2024-10-13

**Authors:** Hui Zhang, Qiang Liu, Lian Chen, Liwei Song, Feng Mao, Wenyong Zhou, Jiantao Li, Zuodong Song, Wang Miao, Yang Shentu

**Affiliations:** ^1^ Shanghai Lung Cancer Center, Shanghai Chest Hospital Shanghai Jiao Tong University School of Medicine Shanghai China; ^2^ Department of Oncology Dongying People's Hospital Dongying Shandong China; ^3^ Rehabilitation Department Shanghai Fifth Rehabilitation Hospital Shanghai China; ^4^ Department of Thoracic Surgery, Shanghai Chest Hospital Shanghai Jiao Tong University School of Medicine Shanghai China; ^5^ Department of Thoracic Surgery The Third people's Hospital of Zhengzhou Zhengzhou China

**Keywords:** overall survival, prognostic factors, recurrence‐free survival, staged bilateral surgery, synchronous multiple primary lung cancer

## Abstract

**Introduction:**

Staged bilateral surgery is widely used to treat synchronous multiple primary lung cancer (SMPLC); however, the prognostic factors for survival outcomes remain unclear. This study aimed to identify prognostic factors and construct a predictive model for overall survival (OS) and recurrence‐free survival (RFS) in patients with SMPLC who underwent staged bilateral surgery.

**Methods:**

The study included 256 patients diagnosed with SMPLC and treated with staged bilateral surgery at our hospital between January 2010 and July 2017. Multivariate Cox proportional‐hazard regression was used to identify prognostic factors for OS and RFS. Additionally, a predictive model was constructed using time‐dependent receiver operating characteristic curves.

**Results:**

Among the 256 patients, 10 (3.95%) succumbed to the disease and 24 (9.41%) experienced recurrence. Smoking (hazard ratio [HR]: 5.128; 95% confidence interval [CI]: 1.442–18.233; *p* = 0.012) and most advanced pathological TNM (pTNM) stage (II + III) (HR: 12.938; 95% CI: 2.650–63.176; *p* = 0.002) were identified as significant predictors of poor OS. A prognostic model was developed for predicting OS, with a 5‐year area under the curve (AUC) of 0.854. Furthermore, most advanced pTNM stage (II + III) was associated with poor RFS (HR: 5.964; 95% CI: 2.669–13.327; *p* < 0.001), and the predictive model exhibited a 5‐year AUC of 0.718 for RFS.

**Conclusion:**

This study revealed that smoking and most advanced pTNM stage were independent prognostic factors associated with poor OS in patients with bilateral SMPLC. Moreover, most advanced pTNM stage was also linked to unfavorable RFS. The developed predictive model demonstrated moderate prognostic performance for both OS and RFS.

## Graphical abstract

This study shows time‐dependent receiver operating characteristic (t‐ROC) curve for OS.

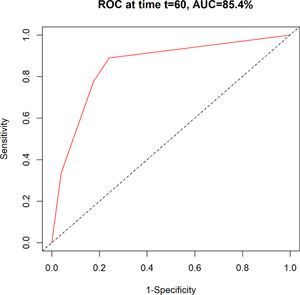

## 1 Introduction

Lung cancer is a prevalent and highly lethal form of cancer worldwide [1]. Multiple primary lung cancer (MPLC) is a distinct subtype of lung cancer characterized by the presence of two or more primary tumors in the lung parenchyma. The incidence of MPLC in lung cancer was 2.0% and range from 0.3% to 6.2%, depending on the diagnostic criteria used and patient population studied [2]. Ground‐glass opacities are commonly observed on high‐resolution computed tomography (CT) scans and have contributed to the increased detection of MPLC due to the widespread use of CT and growing concerns for public health [3, 4]. MPLC can be divided into synchronous and metachronous types based on the occurrence time. Synchronous MPLC (SMPLC) refer to the simultaneous occurrence of MPLC, while metachronous MPLC refer to the occurrence of MPLC at different time points [2, 5]. Treating SMPLC remains challenging, primarily because of the presence of multiple lesions and particularly when lesions are bilateral.

Surgical resection is widely used for SMPLC, particularly video‐assisted thoracoscopic surgery, although the procedure is challenging because the lesions can be located bilaterally in different lobes [6]. Simultaneous bilateral resection is associated with limited surgical experience and a higher risk of postoperative complications. As a result, staged bilateral surgery has been introduced and has gained widespread acceptance in clinical practice. Current guidelines recommend staged bilateral surgery for patients with SMPLC without N2 disease or distant metastases [7]. Numerous studies have investigated and illustrated the therapeutic effects of bilateral surgery for SMPLC [8, 9, 10, 11]. Nonetheless, the prognostic factors specifically related to SMPLC patients treated with staged bilateral surgery have not been well established. Therefore, the primary objective of this study was to identify prognostic factors for overall survival (OS) and recurrence‐free survival (RFS) in SMPLC patients who underwent staged bilateral surgery. Subsequently, we aimed to construct a predictive model to identify high‐risk patients and enhance the prognosis of individuals with SMPLC.

The current study followed the Strengthening the Reporting of Observational Studies in Epidemiology checklist to ensure comprehensive reporting.

## 2 Materials and Methods

### 2.1 Study Design and Patients

Data from 2380 patients with MPLC treated at our hospital between January 2010 and July 2017 were retrospectively collected. The inclusion criteria for patients were as follows: (1) diagnosis of SMPLC based on the 2013 American College of Chest Physicians criteria and the criteria proposed by Martini and Melamed (Table S1) [12, 13]; (2) patients who underwent staged bilateral surgery; (3) confirmation of SMPLC through pathological examination; (4) availability of baseline characteristics and preoperative and clinical data; and (5) the investigated outcomes OS and RFS could obtained. Patients were excluded based on the following criteria: (1) presence of metachronous MPLC; (2) presence of unilateral MPLC; (3) receipt of preoperative radiotherapy, chemotherapy, targeted therapy, or other antitumor treatments; (4) patients with extrathoracic metastases; (5) patients with same bilateral pathological types and mediastinal lymph node metastasis; and (6) unavailability of preoperative and postoperative data. After screening the records of patients, 256 patients with SMPLC who underwent staged bilateral surgery were included for analysis. The study was conducted in accordance with the Declaration of Helsinki (as revised in 2013). The study was approved by the Institutional Review Board of Shanghai Chest Hospital (No. IS23044) and informed consent was obtained from all included patients prior to their participation in the study.

### 2.2 Data Collection

The following data were recruited from electronic medical system, including gender, age, body mass index (BMI), smoking, symptoms, preoperative carcinoembryonic antigen (CEA), preoperative pulmonary function (percentage of forced expiratory volume, carbon monoxide diffusing capacity), surgical approach, type of pulmonary resection, pathology, largest tumor size, number of tumors, postoperative complications, residual nodules, most advanced pathological TNM (pTNM) stage, largest T stage, and highest N stage.

### 2.3 Pre‐Operative Evaluation and Surgical Method

Prior to surgery, all patients underwent a comprehensive preoperative evaluation, which included a chest CT or whole‐body positron emission tomography‐CT scan, abdominal ultrasound or upper abdominal CT scan, brain magnetic resonance imaging (MRI) or brain CT scan, bone scan, and cardiopulmonary function assessment. Bilateral staging surgery was performed on all patients, and the choice of the surgical procedure was based on the preoperative evaluation, considering patient age, cardiopulmonary function, and extent of resection required. Surgical resection options included lobectomy (including combined lobectomy and sleeve lobectomy), sublobar resection (segmentectomy and wedge resection), thoracotomy, video‐assisted thoracoscopic surgery (VATS), and robotic‐assisted thoracoscopic surgery (RATS). In cases where contralateral surgery was required, it was typically performed within 1 year of the initial surgery. During the surgical procedures, all patients underwent systemic mediastinal lymph node dissection or sampling to assess the lymph node involvement.

### 2.4 Follow‐Up

Post‐operative follow‐up consisted of a CT scan every 3 months for the first 2 years and twice a year thereafter. The time interval between the tumors was calculated from the date of the prior tumor resection to the date of detection of the subsequent tumor by imaging. Patients were followed‐up until March 31, 2022. Disease recurrence was classified as either local or distant. Local disease recurrence referred to the reappearance of cancer in the same lobe, or in the hilar or mediastinal lymph nodes. Recurrences occurring in other locations were categorized as distant metastases. Survival time was calculated from the date of the second tumor resection to the date of the last follow‐up or death. The efficacy of cancer treatment was evaluated based on OS, RFS, and rates of local and distant recurrences. OS was defined as the duration from the second tumor resection until death from any cause, while RFS was defined as the duration from the second surgical resection until the occurrence of a recurrence or death. All patients were restaged to assess the improvement in SMPLC according to the 8th TNM staging system.

### 2.5 Statistical Analysis

Categorical data are presented as percentages, the continuous variables conforming to a normal distribution are expressed as mean (standard deviation), while those not conforming to a normal distribution are described using the median and interquartile range to represent the central tendency and dispersion of the data. The prognostic factors for OS and RFS were initially identified using Kaplan–Meier analysis with a log‐rank test, and multivariate Cox proportional hazards regression was performed to identify prognostic factors after adjusting for potential confounding factors. The modeling starts with all variables, and non‐significantly correlated variables with OS and RFS (*p* > 0.05) are removed from the model through a step‐down procedure. The effect estimate was assessed using hazard ratios (HRs) with corresponding 95% confidence intervals (CIs). Subsequently, a time‐dependent receiver operating characteristic (t‐ROC) curve was constructed, and prognostic performance was assessed using the area under the curve (AUC). The inspection level for the identified prognostic factors was two‐sided, and a *p* < 0.05 was regarded as statistically significant. All analyses were performed using IBM SPSS (IBM SPSS Statistics, Chicago, IL, United States, version 26.0) and R software (version 4.0.5; http://www.r‐project.org).

## 3 Results

### 3.1 Enrolled Patients

A total of 31 583 patients underwent surgery for lung cancer in our hospital. Of these, 2380 (7.5%) were diagnosed with MPLC, and 291 patients with bilateral MPLC underwent bilateral surgery. Following the exclusion of 35 patients with metachronous MPLC, history of other cancers, or loss to follow‐up, 256 patients were finally included in the analysis.

### 3.2 Baseline Characteristics

The baseline patient characteristics are presented in Table 1. Of the 256 included patients, 75 (29.30%) were male, and 181 (70.70%) were female. The median age of the included patients was 60.00 (53.00, 64.00) years, with a BMI of 22.90 (20.96, 24.77) kg/m^2^. Most patients were asymptomatic (89.06%), while 24 (9.38%) patients had respiratory‐related symptoms. Twenty‐five (9.77%) patients had a history of smoking. Twenty‐four (9.38%) patients had increased CEA before the first surgical resection procedure. The number of lesions resected per patient was as follows: two lesions (*n* = 118, 46.09%), three to four lesions (*n* = 103, 40.23%), and more than four lesions (*n* = 35, 13.67%). Additionally, 67 (29.00%) patients had residual pulmonary nodules after bilateral surgery. The maximum tumor diameter was less than 2 cm in 187 patients, while it exceeded 2 cm in the remaining 68 patients.

**TABLE 1:** The baseline characteristics of included patients.

Variable	No. (%) or median (range)	Sample size
Gender (%)		256
Male	75 (29.30)	
Female	181 (70.70)	
Age (years)	60.00 (53.00, 64.00)	256
BMI (kg/m^2^)	22.90 (20.96, 24.77)	250
Smoking (%)		256
No	231 (90.23)	
Yes	25 (9.77)	
Symptoms		256
No symptom	228 (89.06)	
Respiratory symptom	24 (9.38)	
Other	4 (1.56)	
Pathology		256
Ad‐Ad	246 (96.09)	
Sq‐Sq	3 (1.17)	
Sq‐Ad	3 (1.17)	
Lc‐Ad	2 (0.78)	
Sq‐Lc	2 (0.78)	
Largest tumor size, cm		255
≤ 2	187 (73.33)	
> 2	68 (26.67)	
Number of tumors		256
≤ 2	118 (46.09)	
> 2, ≤ 4	103 (40.23)	
> 4	35 (13.67)	
Most advanced TNM stage (%)		256
Ia	205 (80.08)	
Ib	33 (12.89)	
IIa	5 (1.95)	
IIb	9 (3.52)	
III	4 (1.56)	
Largest T stage (%)		256
Tis	5 (1.95)	
T1	203 (79.30)	
T2	44 (17.19)	
T3	4 (1.56)	
Highest N stage (%)		256
N0	246 (96.09)	
N1	6 (2.34)	
N2	4 (1.56)	
Preoperative CEA ≥ 5.0 ng/mL	24 (9.38)	256
Preoperative FEV1%	90.90 (84.30, 101.30)	253
Preoperative DLCO (mL/mmHg.min)	18.37 (16.30, 20.41)	252
Surgical approach		256
Open + VATS	27 (10.55)	
Open + Open	3 (1.17)	
VATS + VATS	214 (83.59)	
VATS + RATS	11 (4.30)	
Open + RATS	1 (0.39)	
Type of pulmonary resection		256
Lobectomy + lobectomy	35 (13.67)	
Lobectomy + sublobar	137 (53.52)	
Sublobar + sublobar	84 (32.81)	
Postoperative complications (%)		256
No	188 (73.44)	
Yes	68 (26.56)	
Residual nodules (%)		231
No	164 (71.00)	
Yes	67 (29.00)	
OS	64.00 (56.00, 72.50)	255
OS status (%)		253
Survival	243 (96.05)	
Death	10 (3.95)	
RFS	62.50 (53.75, 72.00)	256
RFS status (%)		255
No	231 (90.59)	
Relapse or metastasis	24 (9.41)	

### 3.3 Surgery and Pathology

Regarding the pathological staging based on the TNM classification, the cases were categorized as stages I (238 cases), II (14 cases), and III (4 cases). Among them, stage Ia was the most common, accounting for 80.08% of patients (*n* = 205). Of the four stage III cases, two had N2 lymphatic metastasis; however, the pathological subtypes of the bilateral lesions were different. T stage was determined based on the largest nodular diameter among the bilateral lesions, resulting in the following classifications: T1 (203 cases), T2 (5 cases), and T3 (4 cases). Only 10 patients had lymphatic metastases, and these cases were classified as SMPLC because of the presence of different pathological types or subtypes in the bilateral lesions.

Various surgical techniques were employed in this study, including open surgery, VATS, and RATS. Among these, bilateral VATS was the most frequently performed (214 cases), while unilateral RATS was the least common (12 cases). Postoperatively, 68 (26.56%) patients experienced postoperative complications, including pneumonia, atelectasis, chylothorax, subcutaneous emphysema, poor wound healing, deep vein thrombosis, and urinary tract infection. However, no deaths occurred postoperatively. Bilateral lobectomy was the least common (35 cases) resection, followed by bilateral sublobar resection was the second common (84 cases). Sublobar resection included segmentectomy, combined segmentectomy, wedge resection, and extended segmentectomy, whereas lobectomy included sleeve resection.

A total of 249 patients had the same bilateral pathological type, which was bilateral lung adenocarcinoma (246 cases). Additionally, 7 patients had bilateral pathologies of different types, with the most common being a combination of squamous and adenocarcinoma.

### 3.4 Overall Survival

**FIGURE 1:**
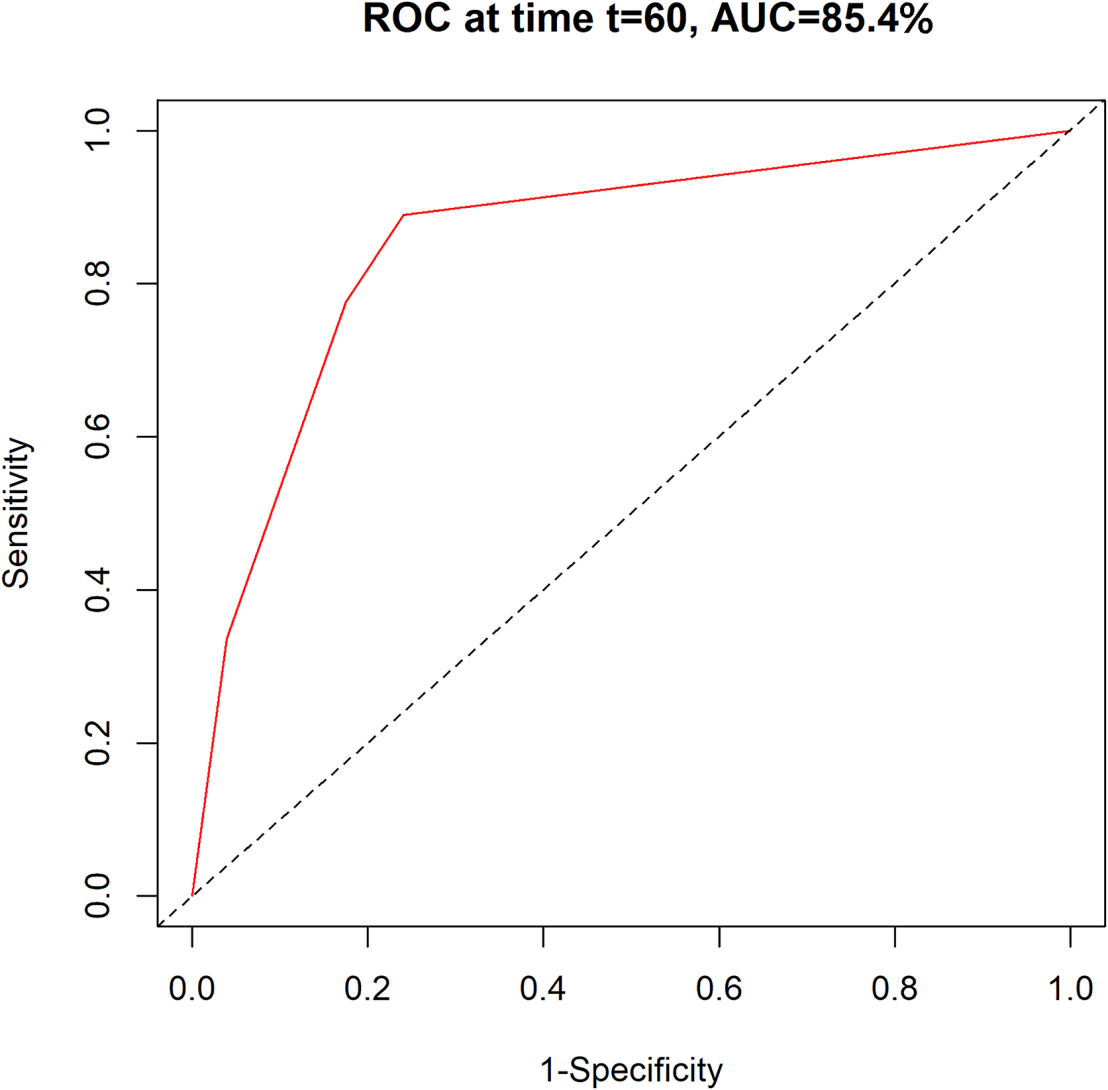
Time‐dependent receiver operating characteristic (t‐ROC) curve for OS.

Ten patients (3.95%) died during follow‐up, and the median OS was 64.00 (56.00, 72.50) months. The 3‐year mortality and 5‐year mortality rates were 2.73% and 3.52%, respectively. Univariate analysis revealed several factors associated with OS. Female sex was associated with better OS compared to male sex (Figure S1). However, smoking, CEA level ≥ 5 ng/mL, surgical approach (VATS + VATS), different pathology, largest tumor size > 2 cm, most advanced pTNM stage (II + III), largest pT stage (T2 + T3), and highest pN stage (1 + 2) were associated with poor OS (Figures S2–S9). After adjusting for potential confounding factors, we found that smoking (HR: 5.128; 95% CI: 1.442–18.233; *p* = 0.012) and the most advanced pTNM stage II + III (HR: 12.938; 95% CI: 2.650–63.176; *p* = 0.002) were associated with poor OS in patients with SMPLC treated with staged bilateral surgery (Table 2). The constructed prognostic model for OS was based on smoking status and the most advanced pTNM stage, with a 5‐year AUC of 0.854, suggesting moderate prognostic performance (Figure 1).

**TABLE 2:** Univariate and multivariate COX proportional hazards regression for OS.

Variable	Univariate		Multivariate	
	HR and 95% CI	*p*	HR and 95% CI	*p*
Gender (%)				
Male	1			
Female	0.044 (0.006–0.344)	0.003		
Age (years)	1.050 (0.971–1.137)	0.222		
BMI (kg/m^2^)	1.145 (0.945–1.388)	0.165		
Smoking (%)				
No	1		1	
Yes	9.490 (2.747–32.787)	< 0.001	5.128 (1.442–18.233)	0.012
Symptoms				
No	1			
Yes	3.325 (0.860–12.858)	0.082		
Pathology				
Same	1			
Different	6.333 (1.343–29.857)	0.020		
Largest tumor size, cm				
≤ 2	1			
> 2	4.197 (1.184–14.873)	0.026		
Number of tumors				
≤ 2	1			
> 2, ≤ 4	0.992 (0.266–3.696)	0.990		
> 4	0.736 (0.085–6.329)	0.780		
Most advanced pTNM stage (%)				
I	1		1	
II + III	17.893 (3.799–84.274)	< 0.001	12.938 (2.650–63.176)	0.002
Largest pT stage (%)				
Tis + T1	1			
T2 + T3	11.384 (2.943–44.027)	< 0.001		
Highest pN stage (%)				
0	1			
1 + 2	6.944 (1.474–32.720)	0.014		
Preoperative CEA, ng/mL				
< 5	1			
≥ 5	6.413 (1.807–22.759)	0.004		
Preoperative FEV1%	0.979 (0.942–1.018)	0.295		
Preoperative DLCO (mL/mmHg.min)	0.992 (0.854–1.154)	0.920		
Surgical approach				
VATS + VATS	1			
Other	7.986 (2.253–28.312)	0.001		
Type of pulmonary resection				
Lobectomy + lobectomy	1			
Lobectomy + sublobar	1.288 (0.150–11.023)	0.817		
Sublobar + sublobar	1.737 (0.194–15.537)	0.622		
Postoperative complications (%)				
No	1			
Yes	1.246 (0.322–4.822)	0.750		
Residual nodules (%)				
No	1			
Yes	0.497 (0.058–4.255)	0.523		

### 3.5 Recurrence‐Free Survival

**FIGURE 2:**
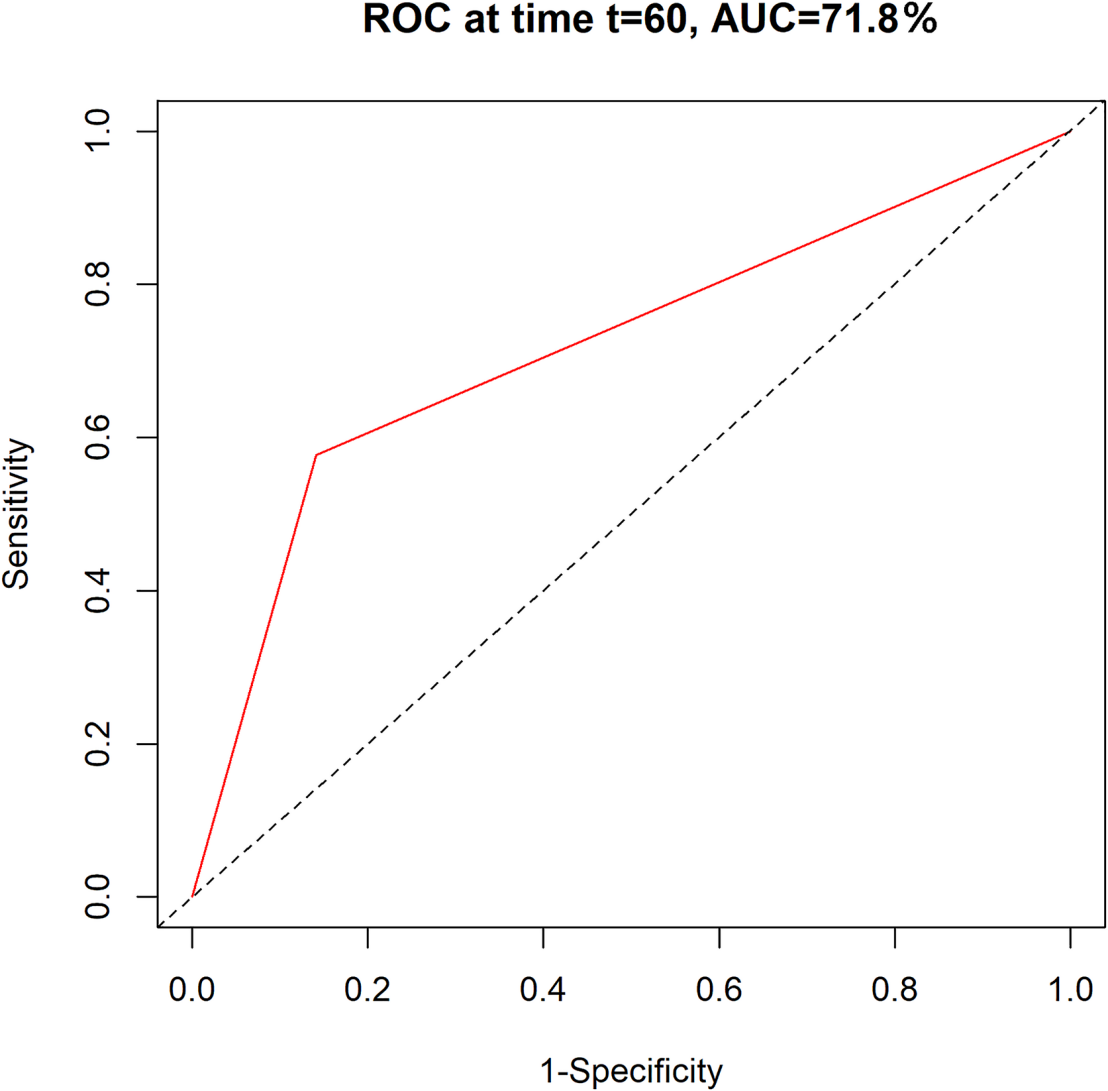
The time‐dependent receiver operating characteristic (t‐ROC) curve for RFS.

A total of 24 patients (9.41%) reported recurrence during follow‐up, and the median RFS was 62.50 (53.75, 72.00) months. The 3‐year recurrence and 5‐year recurrence rates were 5.08% and 8.59%, respectively. Univariate analysis showed that female sex was associated with longer RFS compared to male sex (Figure S10). Increased age, the presence of symptoms, preoperative CEA level ≥ 5 ng/mL, surgical approach (VATS + VATS), largest tumor size > 2 cm, most advanced pTNM stage (II + III), T2 + T3 stage, and N1 + 2 stage (Figures S11–S17) were associated with shorter RFS. After adjusting for potential confounding factors, the multivariate analysis revealed that the most advanced pTNM stage (II + III) was independently associated with poor RFS (HR: 5.964; 95% CI: 2.669–13.327; *p* < 0.001; Table 3). A prognostic model for predicting RFS was constructed based on this factor, and it showed a 5‐year area under the curve (AUC) of 0.718 (Figure 2).

**TABLE 3:** Univariate and multivariate COX proportional hazards regression for RFS.

Variable	Univariate		Multivariate	
	HR and 95% CI	*p*	HR and 95% CI	*p*
Gender (%)				
Male	1			
Female	0.447 (0.200–0.998)	0.049		
Age (years)	1.055 (1.002–1.110)	0.042		
BMI (kg/m^2^)	1.050 (0.920–1.198)	0.470		
Smoking (%)				
No	1			
Yes	2.007 (0.686–5.874)	0.204		
Symptoms				
No	1			
Yes	2.869 (1.139–7.229)	0.025		
Pathology				
Same	1			
Different	1.087 (0.147–8.053)	0.935		
Largest tumor size, cm				
≤ 2	1			
> 2	3.469 (1.554–7.744)	0.002		
Number of tumors				
≤ 2	1			
> 2, ≤ 4	0.786 (0.321–1.922)	0.597		
> 4	1.184 (0.381–3.685)	0.771		
Most advanced pTNM stage (%)				
I	1		1	
II + III	5.964 (2.669–13.327)	<0.001	5.964 (2.669–13.327)	<0.001
Largest pT stage (%)				
Tis + T1	1			
T2 + T3	5.460 (2.450–12.168)	<0.001		
Highest pN stage (%)				
0	1			
1 + 2	9.726 (3.616–26.158)	<0.001		
Preoperative CEA, ng/mL				
< 5	1			
≥ 5	2.922 (1.089–7.841)	0.033		
Preoperative FEV1%	0.989 (0.965–1.015)	0.410		
Preoperative DLCO (mL/mmHg.min)	0.966 (0.877–1.064)	0.484		
Surgical approach				
VATS + VATS	1			
Other	3.481 (1.522–7.961)	0.003		
Type of pulmonary resection				
Lobectomy + lobectomy	1			
Lobectomy + sublobar	0.764 (0.246–2.368)	0.640		
Sublobar + sublobar	0.830 (0.250–2.759)	0.762		
Postoperative complications (%)				
No	1			
Yes	1.422 (0.609–3.325)	0.416		
Residual nodules (%)				
No	1			
Yes	1.142 (0.465–2.801)	0.773		

## 4 Discussion

The study aimed to investigate the prognostic factors for OS and RFS and to construct a multifactorial prognostic model for patients with SMPLC who underwent staged bilateral surgery. Retrospective data were collected from 256 patients with SMPLC who underwent staged bilateral surgery and reported the 5‐year OS and RFS were 96.05% and 90.59%, respectively, this excellent outcomes could explained by the high prevalence of stage I patients in this study, accounting for 93.0% of the cases. A considerable proportion of stage I patients exhibited multiple ground glass opacities on CT scans. Generally, lung cancer patients with ground glass components tend to have a better prognosis, which may have contributed to the overall survival rate observed in our study [14]. Moreover, smoking and the most advanced pTNM stage II + III were associated with poor OS, and the prognostic model based on these factors had moderate performance for predicting OS. Furthermore, the most advanced pTNM stage (II + III) was associated with poor RFS, and a model with mild prognostic performance for predicting RFS. These results may provide a simple prognostic tool to identify patients at high risk of death or relapse, and allow the implementation of effective intervention strategies to improve the prognosis in these patients.

In a review, Lv et al. [15] retrospectively collected 164 patients with synchronous multifocal lung cancers and found that tumor size, lymphatic metastases, and histologic differentiation were significantly associated with OS and progression‐free survival, whereas TNM stage did not affect patient survival. Moreover, the authors reported a 5‐year survival rate of 100% for patients with a tumor size of 0.8 cm or smaller [15]. Ma et al. [16] identified 206 patients with SMPLC and constructed a CT‐based prognostic score that contained six chest CT parameters, suggesting that the constructed model had a higher prediction performance for OS and disease‐free survival. However, the identified prognostic factors did not include perioperative data, and the study population did not focus on patients with SMPLC treated with staged bilateral surgery [16]. Therefore, the current study was conducted to address these gaps by identifying prognostic factors and constructing a prognostic model for OS and RFS specifically in patients with SMPLC treated with staged bilateral surgery. The study aimed to incorporate patient characteristics and preoperative, perioperative, and postoperative data to develop a comprehensive prognostic model.

Our study found that smoking and the most advanced pTNM stage (II + III) were associated with poor OS, while the most advanced pTNM stage (II + III) was associated with poor RFS in patients with SMPLC treated with staged bilateral surgery. Various studies have demonstrated that smoking status may affect the prognosis of lung cancer [17, 18, 19], and the reasons included: (1) smoking can accelerate tumor growth and spread, making lung cancer patients more likely to experience disease progression; (2) smoking can reduce the response of lung cancer patients to treatments such as surgery, chemotherapy, and radiation therapy. Smoking may lead to decreased treatment tolerance and increased risks of complications; (3) smoking can increase the recurrence rate of lung cancer patients, making tumors more likely to reappear or spread to other areas, affecting the long‐term survival of patients; and (4) smoking increases the risk of surgical complications such as postoperative infections and delayed healing, affecting the patient's recovery and prognosis after surgery [20]. Furthermore, advanced pTNM stage (II + III) was associated with poor OS and RFS. Studies have demonstrated that the size and invasion of nearby tissues of primary tumors are determined by the T category [21]. There was a negative association between tumor size and the prognosis of SMPLC, and a high T category was significantly associated with lymph node metastasis, which is significantly associated with relapse and OS [22]. Furthermore, the maximum diameter of the lesion or the highest stage can be determined using the pTNM stage, which is significantly associated with SMPLC prognosis [23]. Finally, we constructed a prognostic model for OS and RFS in patients with SMPLC treated with staged bilateral surgery. The prognostic performance of the model for OS, with a 5‐year AUC of 0.854, suggests that diagnostic performance was moderate. Therefore, smoking cessation should be recommended for patients with SMPLC to improve OS [24]. Moreover, patients with advanced pTNM stages II and III should be cautiously monitored, and additional effective treatment strategies should be applied to improve prognosis.

Several significant prognostic factors were observed in the univariate analyses, including sex, age, symptoms, preoperative CEA level, surgical approach, pathology, tumor size, largest pT stage, and highest pN stage. Several reasons could explained these results: (1) the improved prognosis in female patients may be attributed to a younger age distribution, a greater prevalence of adenocarcinoma histologic subtype, and hormonal factors [25]; (2) age and symptoms are significantly associated with progression and severity of disease, which could affect SMPLC prognosis; (3) preoperative CEA is correlated with the degree of malignancy in SMPLC, and the progression of disease could affected [26]; (4) the surgical approach on the basis of preoperative evaluation, and the considering factors included patient's age, cardiopulmonary function, and the extent of resection required, thus the prognosis of SMPLC patients could affected; (5) adenocarcinoma was the main pathological type in early stage SMPLC with a better prognosis [23]; and (6) tumor size, largest pT stage, and highest pN stage are significantly associated with advanced pTNM stage, affecting prognosis. In our study, multivariate analyses did not reveal a significant prognostic role of the above factors on OS and RFS after adjusting for potential confounding factors, which could be explained by the fact that the number of deaths or relapses was lower than expected, and longer follow‐up periods are required to obtain sufficient power to detect potential significant associations.

This study had several limitations. First, this was a retrospective cohort study, and the available outcomes could have been affected by selection and recall biases. Second, although all patients underwent staged bilateral surgery, the type of resection differed among patients, which could have affected the prognosis of SMPLC, we need to conduct exploratory analysis based on the type of resection in future studies. Third, the follow‐up period in this study showed lower rates of OS and RFS, considering that the main population in this study all suffered from early‐stage lung cancer, therefore, longer follow‐up time is needed to confirm the study results. Fourth, the postoperative treatment strategy for the patient was not taken into consideration, which will affect the patient's prognosis. Finally, the constructed prognostic model was not verified by external validation, and its reliability should be assessed in external cohorts.

This study examined predictive markers for OS and RFS in patients with SMPLC who underwent staged bilateral surgery. Smoking and advanced pTNM stages II + III were substantially associated with poor OS, whereas poor RFS was significantly related to advanced pTNM stages II + III. Our model demonstrated moderate performance for predicting OS and mild prognostic performance for RFS.

## Author Contributions

All authors are accountable for ensuring the integrity and precision of the data presented in this study. The conceptualization of the research was carried out by H. Zhang and Y. Shentu. The methodology was developed by H. Zhang, Z. Song, L. Song, W. Miao, and L. Chen. The formal analysis and investigation were conducted by Q. Liu, L. Chen, F. Mao, and W. Zhou. The initial draft of the manuscript was prepared by H. Zhang. The review and editing of the manuscript were performed by Y. Shentu, Q. Liu, J. Li, and L. Chen. The acquisition of funding was facilitated by H. Zhang. The overall supervision of the research project was overseen by Y. Shentu.

## Ethics Statement

The study protocol was approved by the Institutional Review Board of Shanghai Chest Hospital (No. IS23044), and the study adhered to the principles outlined in the Declaration of Helsinki.

## Consent

Informed consent was obtained from all included patients prior to their participation in the study.

## Conflicts of Interest

The authors declare no conflicts of interest.

## Supporting information


**Table S1.** The criteria for diagnosis of SMPLC.
**Figure S1.** Kaplan–Meier analysis with log‐rank test for the role of sex with OS (female vs. male: HR: 0.044; 95% CI: 0.006–0.344; *p* = 0.003). The horizontal axis is in months, and the vertical axis is in percentage.
**Figure S2.** Kaplan–Meier analysis with log‐rank test for the role of smoking with OS (yes vs. no: HR: 9.490; 95% CI: 2.747–32.787; *p* < 0.001). The horizontal axis is in months, and the vertical axis is in percentage.
**Figure S3.** Kaplan–Meier analysis with log‐rank test for the role of preoperative CEA with OS (≥ 5 vs. < 5 ng/mL: HR: 6.413; 95% CI: 1.807–22.759; *p* = 0.004). The horizontal axis is in months, and the vertical axis is in percentage.
**Figure S4.** Kaplan–Meier analysis with log‐rank test for the role of surgical approach with OS (other vs. VATS+VATS: HR: 7.986; 95% CI: 2.253–28.312; *p* = 0.001). The horizontal axis is in months, and the vertical axis is in percentage.
**Figure S5.** Kaplan–Meier analysis with log‐rank test for the role of pathology with OS (different vs. same: HR: 6.333; 95% CI: 1.343–29.857; *p* = 0.020). The horizontal axis is in months, and the vertical axis is in percentage.
**Figure S6.** Kaplan–Meier analysis with log‐rank test for the role of largest tumor size with OS (>2 vs. ≤ 2: HR: 4.197; 95% CI: 1.184–14.873; *p* = 0.026). The horizontal axis is in months, and the vertical axis is in percentage.
**Figure S7.** Kaplan–Meier analysis with log‐rank test for the role of most advanced TNM stage with OS (II + III vs. I: HR: 17.893; 95% CI: 3.799–84.274; *p* < 0.001). The horizontal axis is in months, and the vertical axis is in percentage.
**Figure S8.** Kaplan–Meier analysis with log‐rank test for the role of largest T stage with OS (T3 + T4 vs. T1 + T2: HR: 11.384; 95% CI: 2.943–44.027; *p* < 0.001). The horizontal axis is in months, and the vertical axis is in percentage.
**Figure S9.** Kaplan–Meier analysis with log‐rank test for the role of highest N stage with OS (1 + 2 vs. 0: HR: 6.944; 95% CI: 1.474–32.720; *p* = 0.014). The horizontal axis is in months, and the vertical axis is in percentage.
**Figure S10.** Kaplan–Meier analysis with log‐rank test for the role of sex with RFS (female vs. male: HR: 0.447; 95% CI: 0.200–0.998; *p* = 0.049). The horizontal axis is in months, and the vertical axis is in percentage.
**Figure S11.** Kaplan–Meier analysis with log‐rank test for the role of symptoms with RFS (yes vs. no: HR: 2.869; 95% CI: 1.139–7.229; *p* = 0.025). The horizontal axis is in months, and the vertical axis is in percentage.
**Figure S12.** Kaplan–Meier analysis with log‐rank test for the role of preoperative CEA with RFS (≥ 5 vs. < 5 ng/mL: HR: 2.922; 95% CI: 1.089–7.841; *p* = 0.033). The horizontal axis is in months, and the vertical axis is in percentage.
**Figure S13.** Kaplan–Meier analysis with log‐rank test for the role of surgical approach with RFS (other vs. VATS+VATS: HR: 3.481; 95% CI: 1.522–7.961; *p* = 0.003). The horizontal axis is in months, and the vertical axis is in percentage.
**Figure S14.** Kaplan–Meier analysis with log‐rank test for the role of largest tumor size with RFS (>2 vs. ≤ 2: HR: 3.469; 95% CI: 1.554–7.744; *p* = 0.002). The horizontal axis is in months, and the vertical axis is in percentage.
**Figure S15.** Kaplan–Meier analysis with log‐rank test for the role of most advanced TNM stage with RFS (II + III vs. I: HR: 5.964; 95% CI: 2.669–13.327; *p* < 0.001). The horizontal axis is in months, and the vertical axis is in percentage.
**Figure S16.** Kaplan–Meier analysis with log‐rank test for the role of largest T stage with RFS (3 + 4 vs. 1 + 2: HR: 5.460; 95% CI: 2.450–12.168; *p* < 0.001). The horizontal axis is in months, and the vertical axis is in percentage.
**Figure S17.** Kaplan–Meier analysis with log‐rank test for the role of highest N stage with RFS (1 + 2 vs. 0: HR: 9.726; 95% CI: 3.616–26.158; *p* < 0.001). The horizontal axis is in months, and the vertical axis is in percentage.

## Data Availability

The datasets used and/or analyzed during the current study are available from the corresponding author on reasonable request.
