# Supplementary material for: Identification of the Prognostic Factors for Synchronous Multiple Primary Lung Cancer Treated With Staged Bilateral Surgery
Source: Clin Respir J. 2024 Oct 13;18(10):e70017. doi: 10.1111/crj.70017 (PMC11471348; doi:10.1111/crj.70017)
Supplement: Supplementary file 1 — Table S1. The criteria for diagnosis of SMPLC. Figure S1. Kaplan–Meier analysis with log‐rank test for the role of sex with OS (female vs. male: HR: 0.044; 95% CI: 0.006–0.344; p = 0.003). The horizontal axis is in months, and the vertical axis is in percentage. Figure S2. Kaplan–Meier analysis with log‐rank test for the role of smoking with OS (yes vs. no: HR: 9.490; 95% CI: 2.747–32.787; p < 0.001). The horizontal axis is in months, and the vertical axis is in percentage. Figure S3. Kaplan–Meier analysis with log‐rank test for the role of preoperative CEA with OS (≥ 5 vs. < 5 ng/mL: HR: 6.413; 95% CI: 1.807–22.759; p = 0.004). The horizontal axis is in months, and the vertical axis is in percentage. Figure S4. Kaplan–Meier analysis with log‐rank test for the role of surgical approach with OS (other vs. VATS+VATS: HR: 7.986; 95% CI: 2.253–28.312; p = 0.001). The horizontal axis is in months, and the vertical axis is in percentage. Figure S5. Kaplan–Meier analysis with log‐rank test for the role of pathology with OS (different vs. same: HR: 6.333; 95% CI: 1.343–29.857; p = 0.020). The horizontal axis is in months, and the vertical axis is in percentage. Figure S6. Kaplan–Meier analysis with log‐rank test for the role of largest tumor size with OS (>2 vs. ≤ 2: HR: 4.197; 95% CI: 1.184–14.873; p = 0.026). The horizontal axis is in months, and the vertical axis is in percentage. Figure S7. Kaplan–Meier analysis with log‐rank test for the role of most advanced TNM stage with OS (II + III vs. I: HR: 17.893; 95% CI: 3.799–84.274; p < 0.001). The horizontal axis is in months, and the vertical axis is in percentage. Figure S8. Kaplan–Meier analysis with log‐rank test for the role of largest T stage with OS (T3 + T4 vs. T1 + T2: HR: 11.384; 95% CI: 2.943–44.027; p < 0.001). The horizontal axis is in months, and the vertical axis is in percentage. Figure S9. Kaplan–Meier analysis with log‐rank test for the role of highest N stage with OS (1 + 2 vs. 0: HR: 6.944; 95% CI: 1.474 [file CRJ-18-e70017-s001.docx]

Table S1. the criteria for diagnosis of SMPLC

| I. Tumors that are separate and individual |
| --- |
| II. Different histology |
| III. Same histological type, if:  a: Tumors arise from carcinoma in situ;  b: Tumors with different histologic subtypes;  c: Tumors have different molecular genetic characteristics;  d: Tumors in different segments and lobes, without mediastinal lymph node metastasis or systemic metastases. |

Modified from Martini and Melamed, and the 2013 ACCP


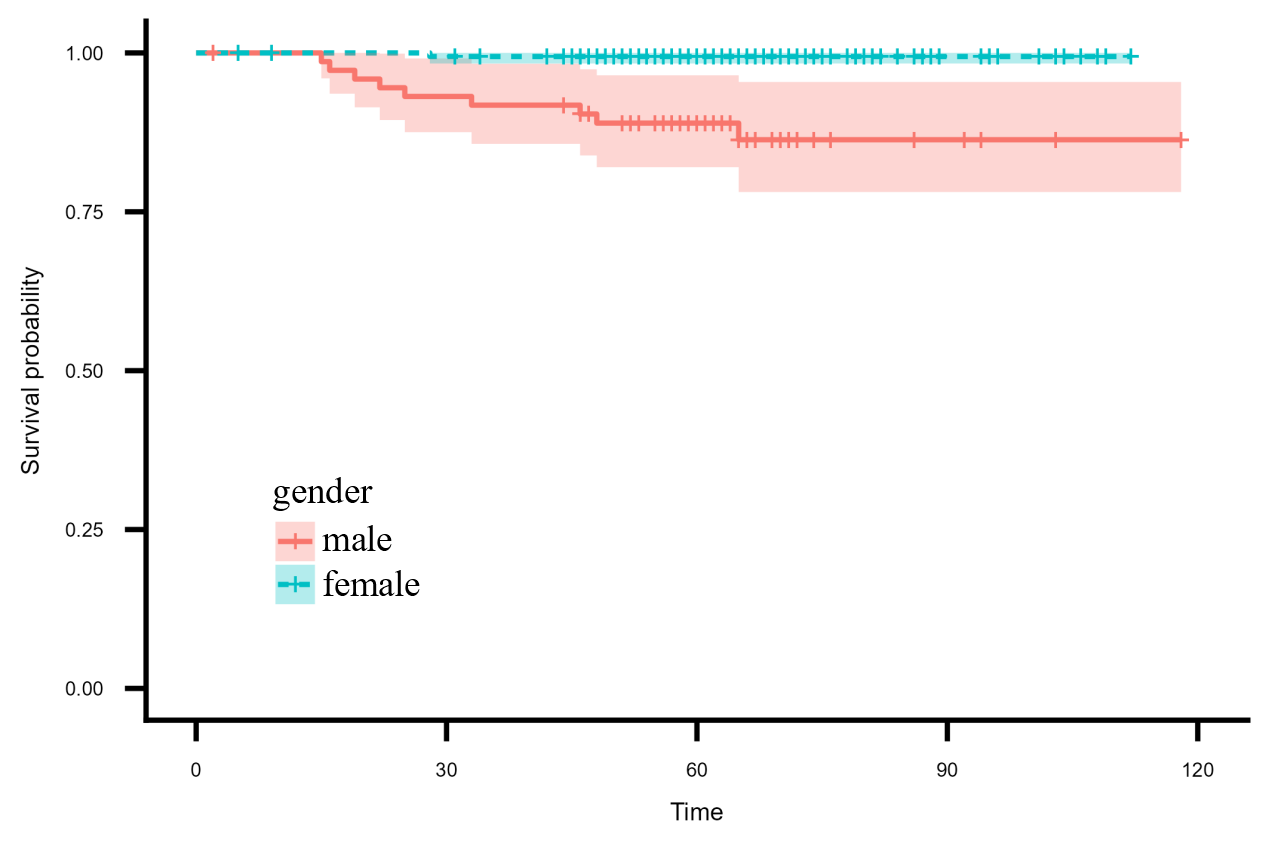


Figure S1. Kaplan-Meier analysis with log-rank test for the role of sex with OS (female vs male: HR: 0.044; 95%CI: 0.006-0.344; P=0.003). The horizontal axis is in months, and the vertical axis is in percentage.


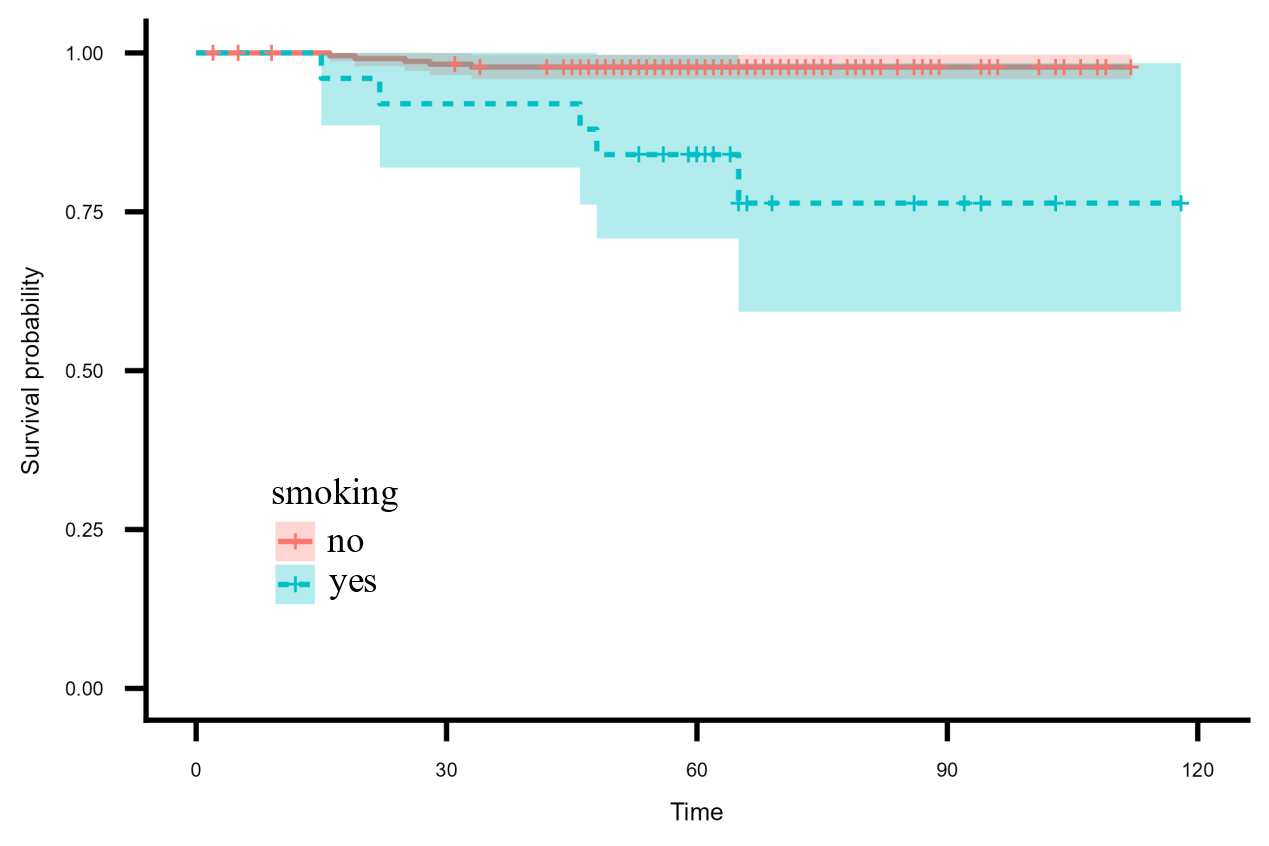


Figure S2. Kaplan-Meier analysis with log-rank test for the role of smoking with OS (yes vs no: HR: 9.490; 95%CI: 2.747-32.787; P<0.001). The horizontal axis is in months, and the vertical axis is in percentage.


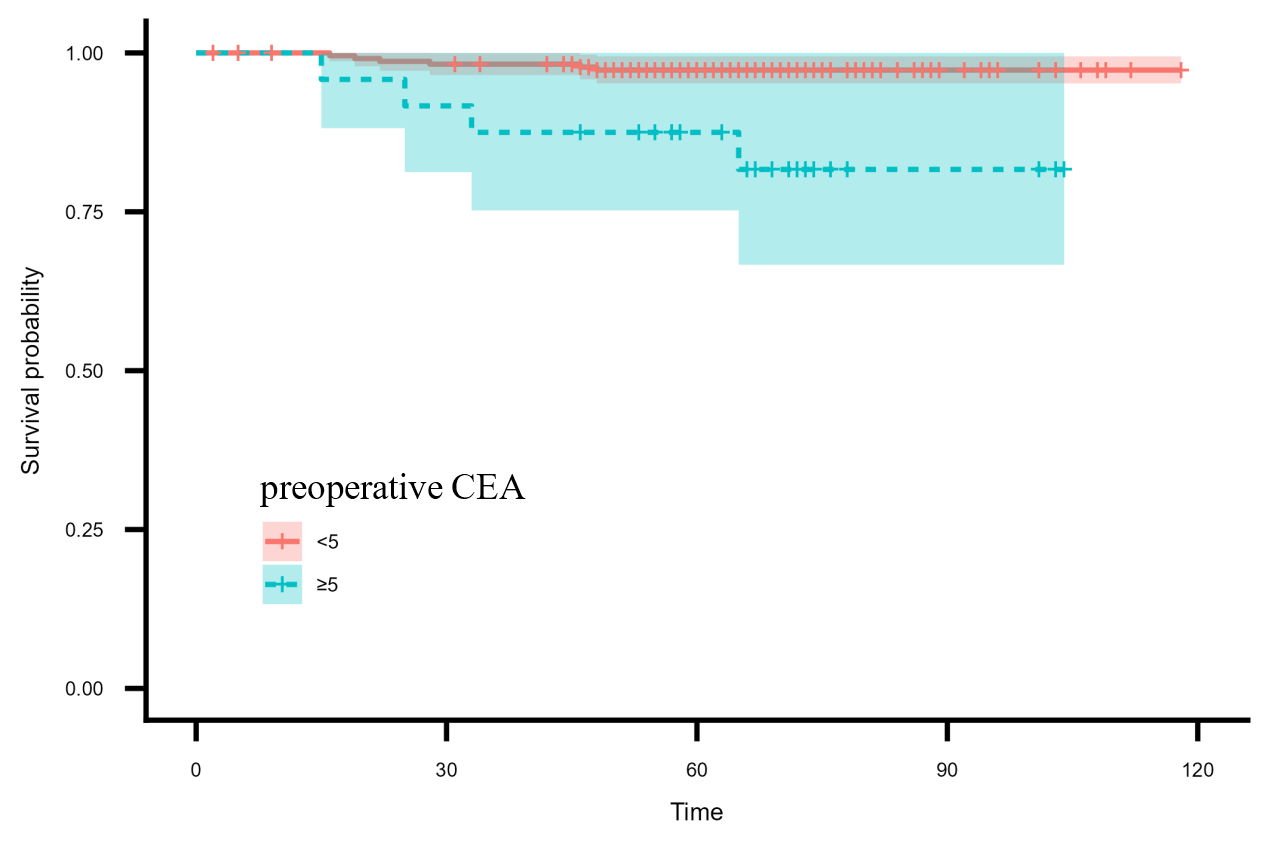


<5

≥5

Figure S3. Kaplan-Meier analysis with log-rank test for the role of preoperative CEA with OS (≥ 5 vs < 5 ng/ml: HR: 6.413; 95%CI: 1.807-22.759; P=0.004). The horizontal axis is in months, and the vertical axis is in percentage.


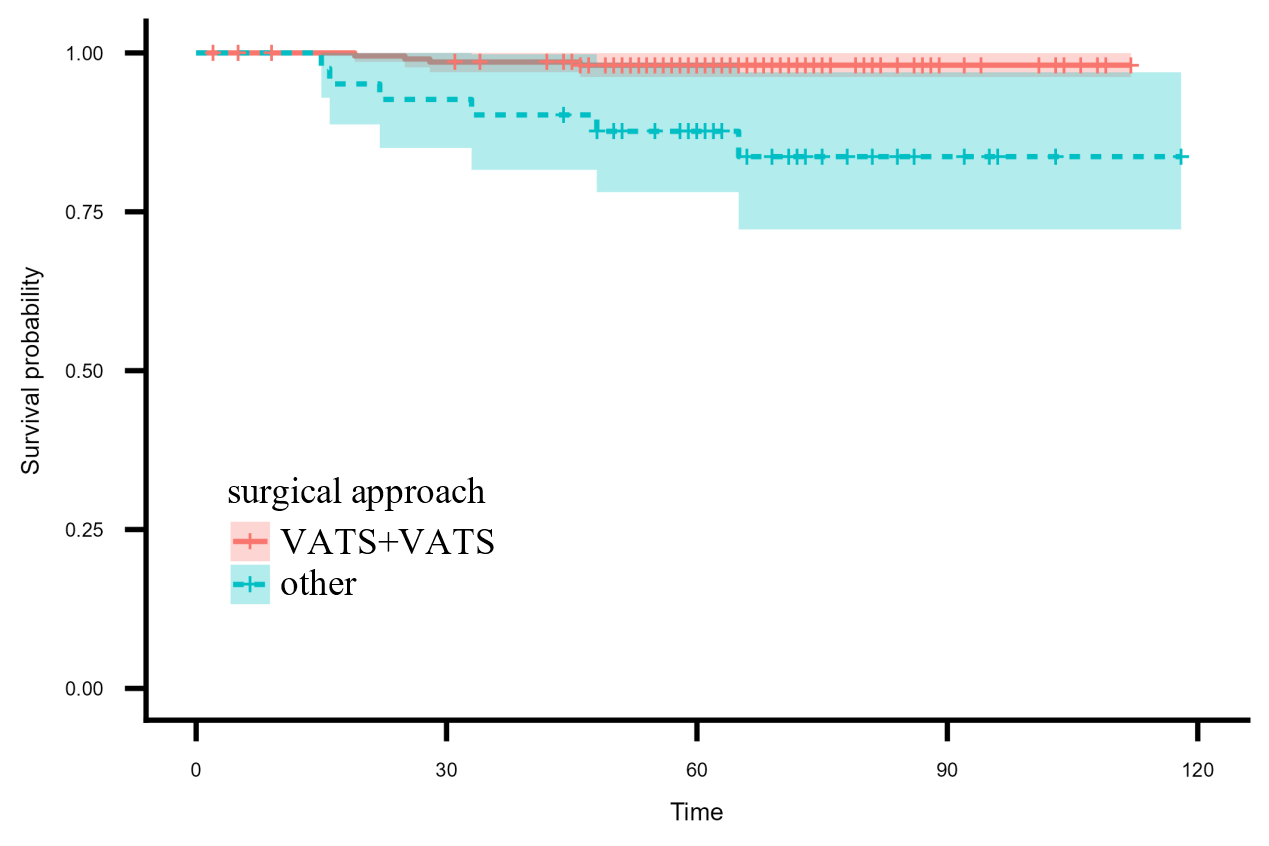


Figure S4. Kaplan-Meier analysis with log-rank test for the role of surgical approach with OS (other vs VATS+VATS: HR: 7.986; 95%CI: 2.253-28.312; P=0.001). The horizontal axis is in months, and the vertical axis is in percentage.


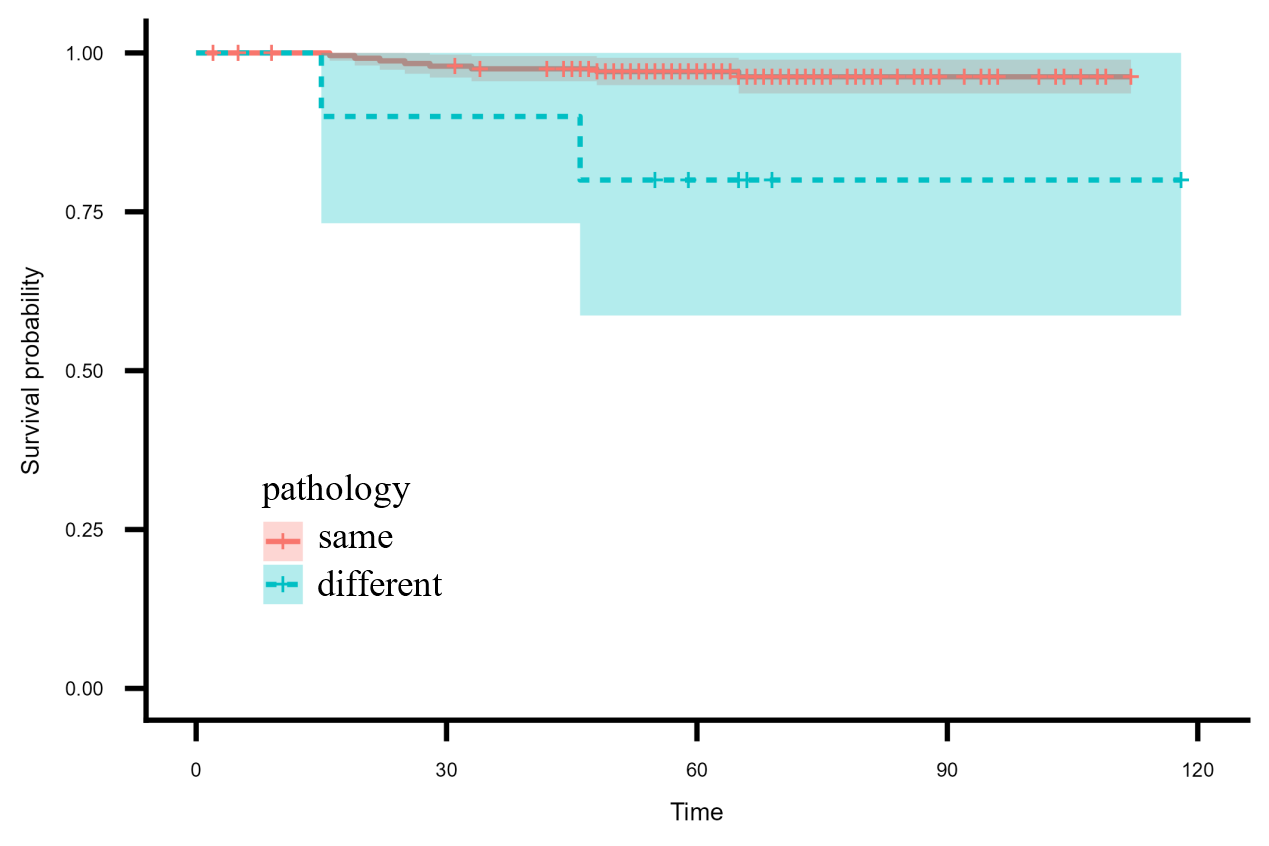


Figure S5. Kaplan-Meier analysis with log-rank test for the role of pathology with OS (different vs same: HR: 6.333; 95%CI: 1.343-29.857; P=0.020). The horizontal axis is in months, and the vertical axis is in percentage.


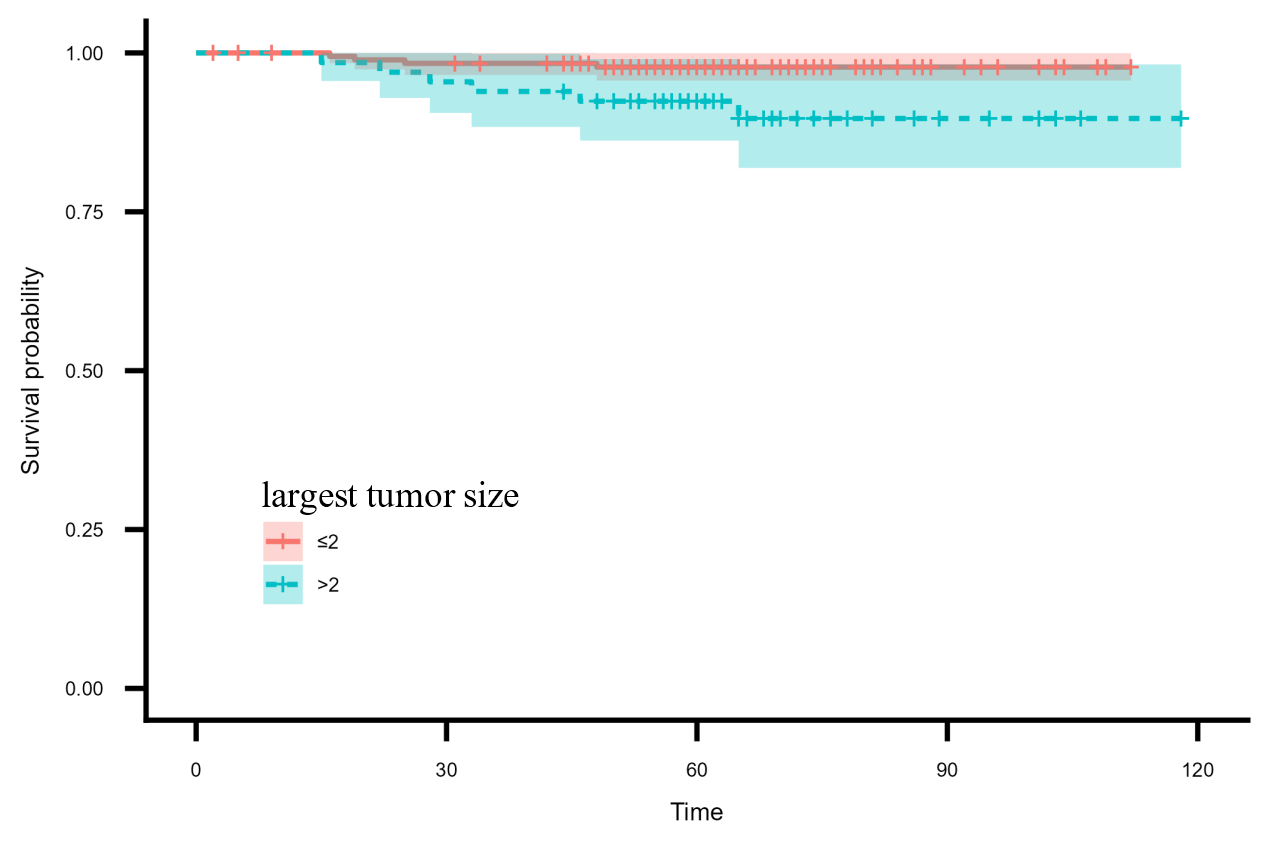


largest tumor size

≤2

>2

Figure S6. Kaplan-Meier analysis with log-rank test for the role of largest tumor size with OS (>2 vs ≤2: HR: 4.197; 95%CI: 1.184-14.873; P=0.026). The horizontal axis is in months, and the vertical axis is in percentage.


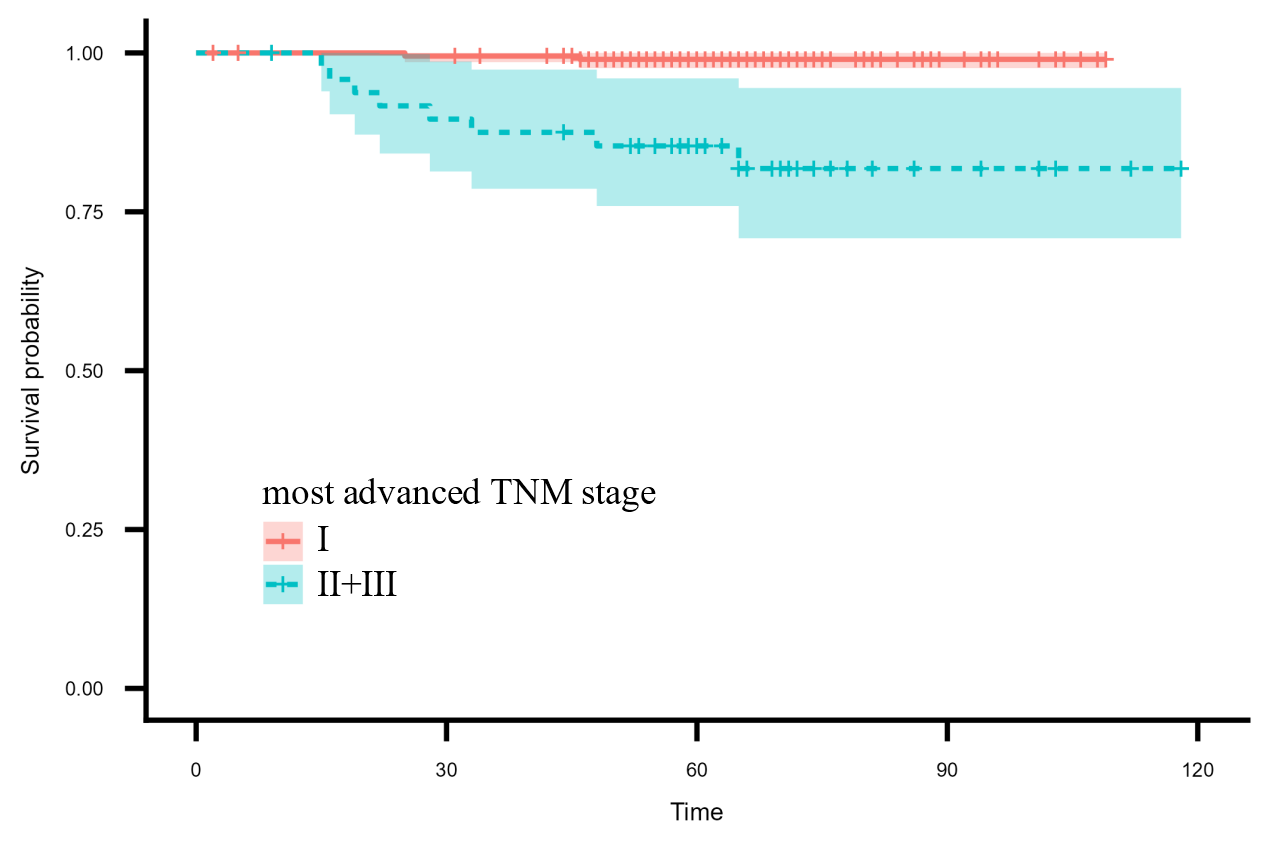


Figure S7. Kaplan-Meier analysis with log-rank test for the role of most advanced TNM stage with OS (II+III vs I: HR: 17.893; 95%CI: 3.799-84.274; P<0.001). The horizontal axis is in months, and the vertical axis is in percentage.


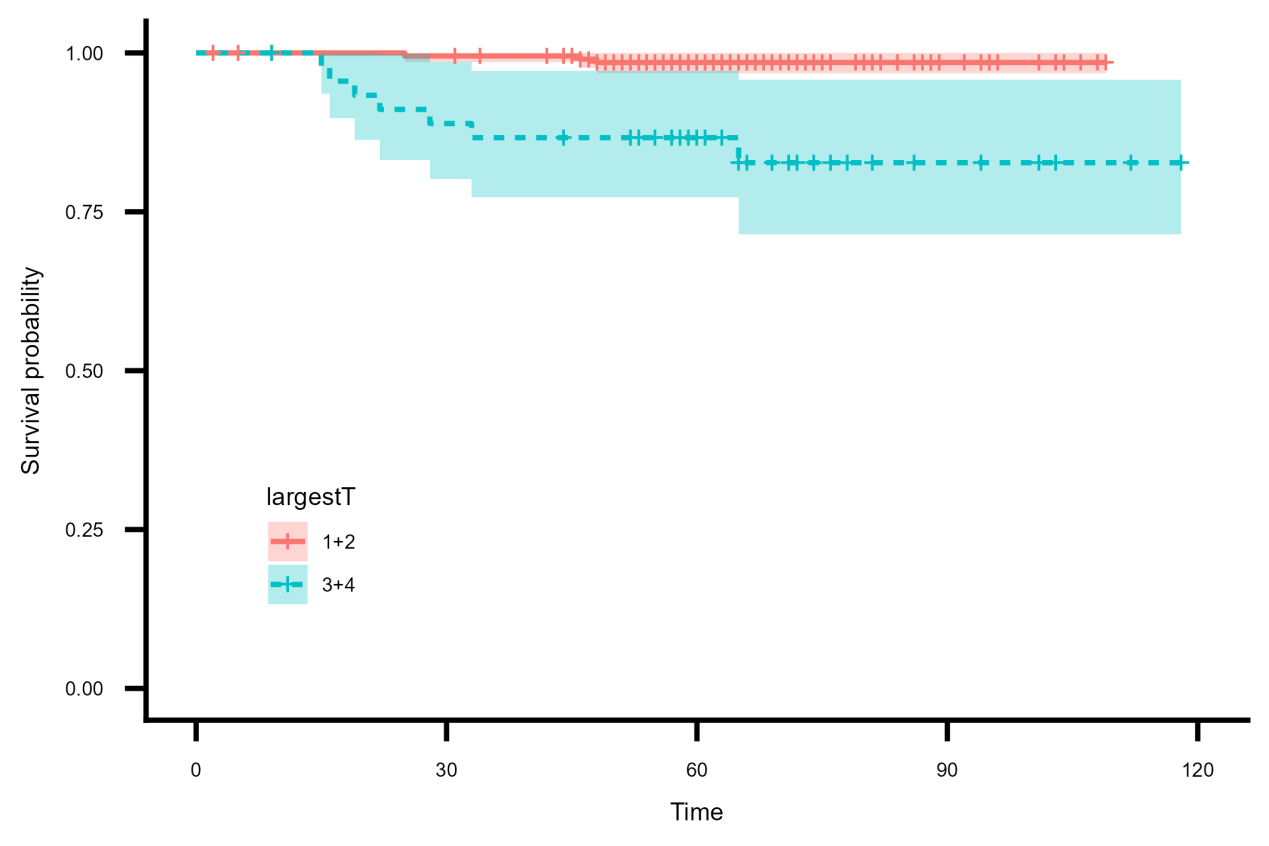


1+2

3+4

largestT

Figure S8. Kaplan-Meier analysis with log-rank test for the role of largest T stage with OS (T3+T4 vs T1+T2: HR: 11.384; 95%CI: 2.943-44.027; P<0.001). The horizontal axis is in months, and the vertical axis is in percentage.


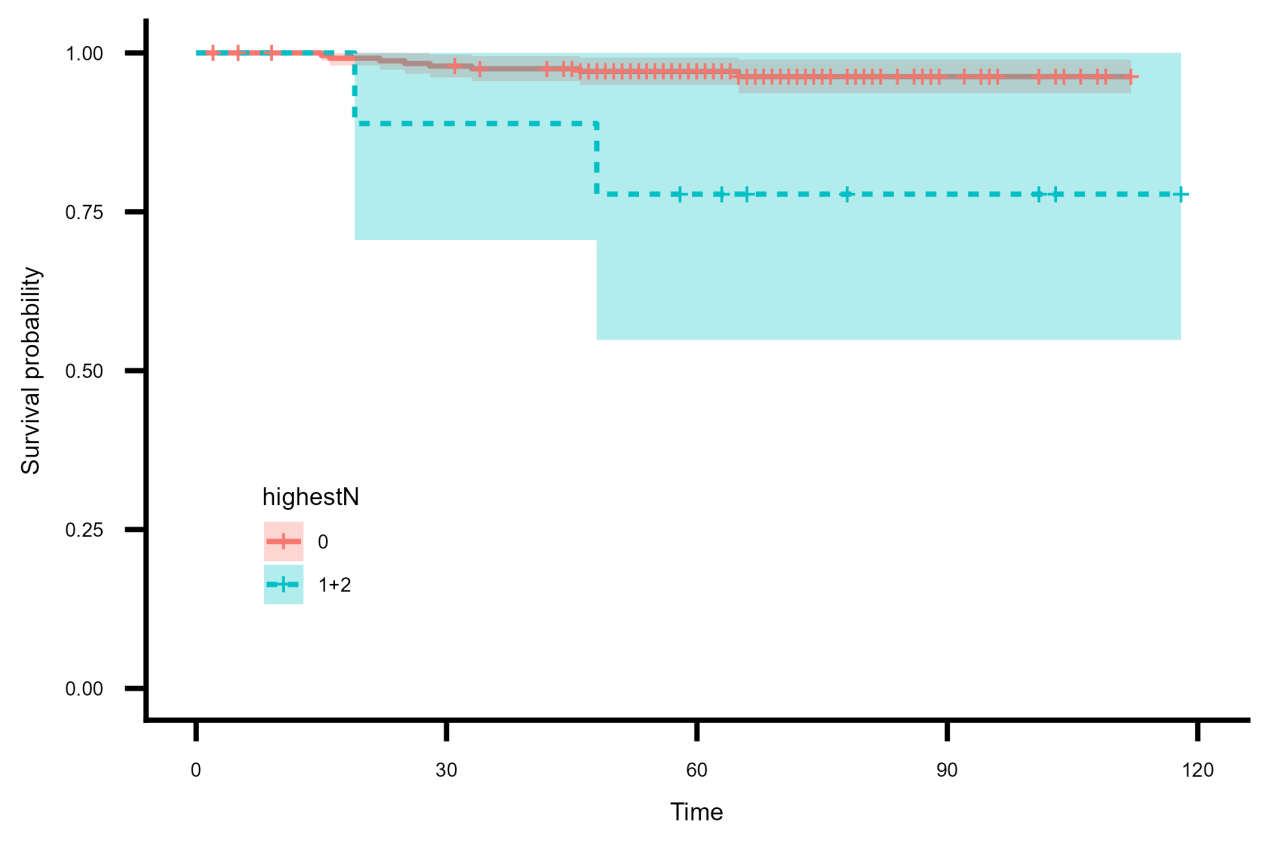


highestN

0

1+2

Figure S9. Kaplan-Meier analysis with log-rank test for the role of highest N stage with OS (1+2 vs 0: HR: 6.944; 95%CI: 1.474-32.720; P=0.014). The horizontal axis is in months, and the vertical axis is in percentage.


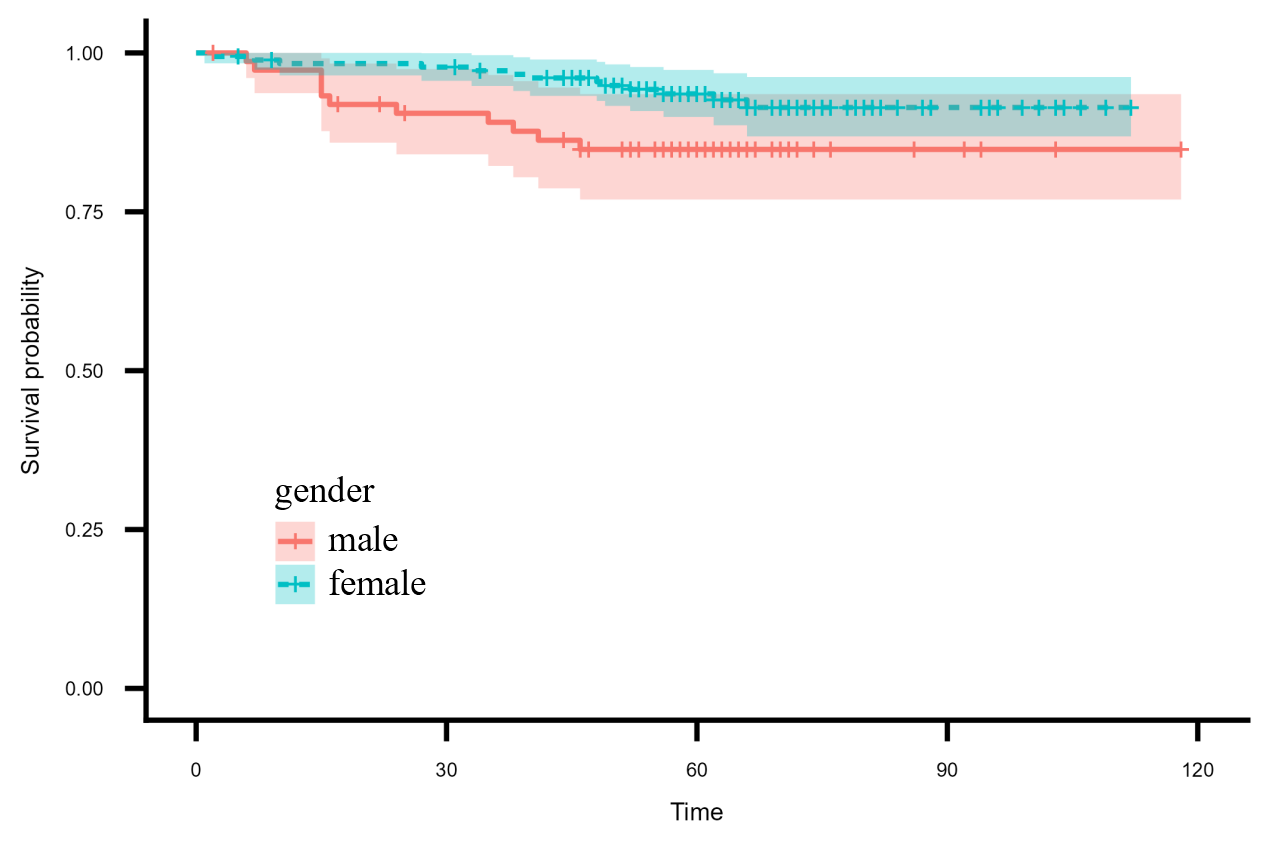


Figure S10. Kaplan-Meier analysis with log-rank test for the role of sex with RFS (female vs male: HR: 0.447; 95%CI: 0.200-0.998; P=0.049). The horizontal axis is in months, and the vertical axis is in percentage.


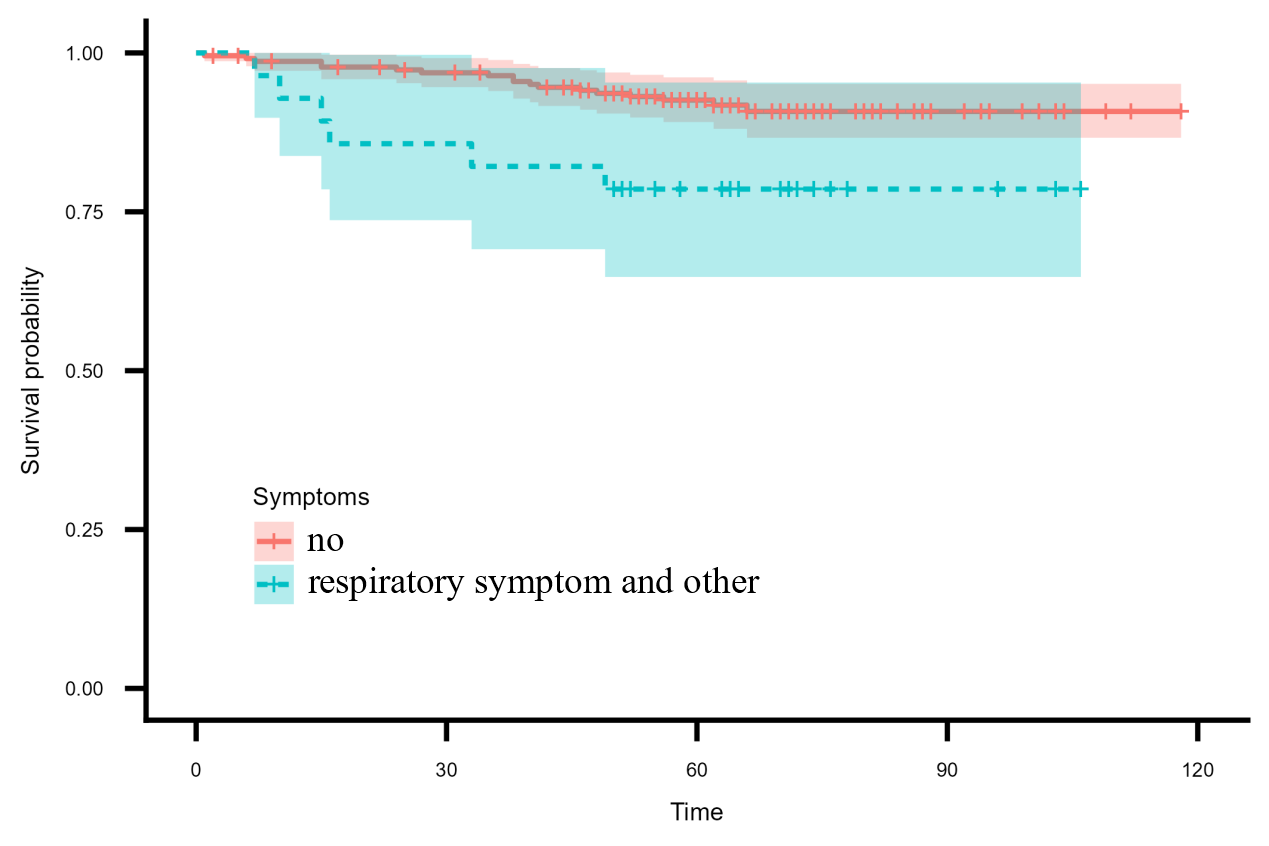


symptoms

Figure S11. Kaplan-Meier analysis with log-rank test for the role of symptoms with RFS (yes vs no: HR: 2.869; 95%CI: 1.139-7.229; P=0.025). The horizontal axis is in months, and the vertical axis is in percentage.


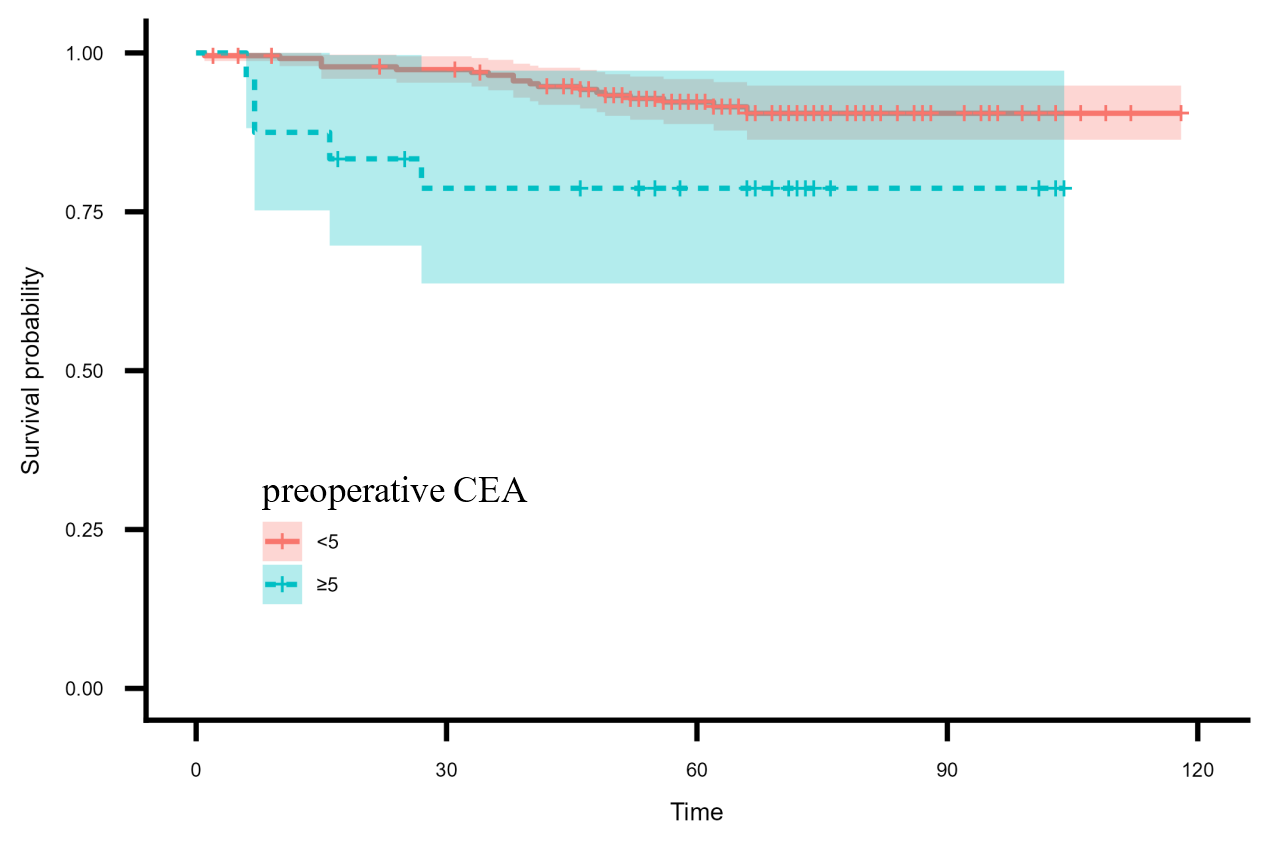


<5

≥5

preoperative CEA

Figure S12. Kaplan-Meier analysis with log-rank test for the role of preoperative CEA with RFS (≥ 5 vs < 5 ng/ml: HR: 2.922; 95%CI: 1.089-7.841; P=0.033). The horizontal axis is in months, and the vertical axis is in percentage.


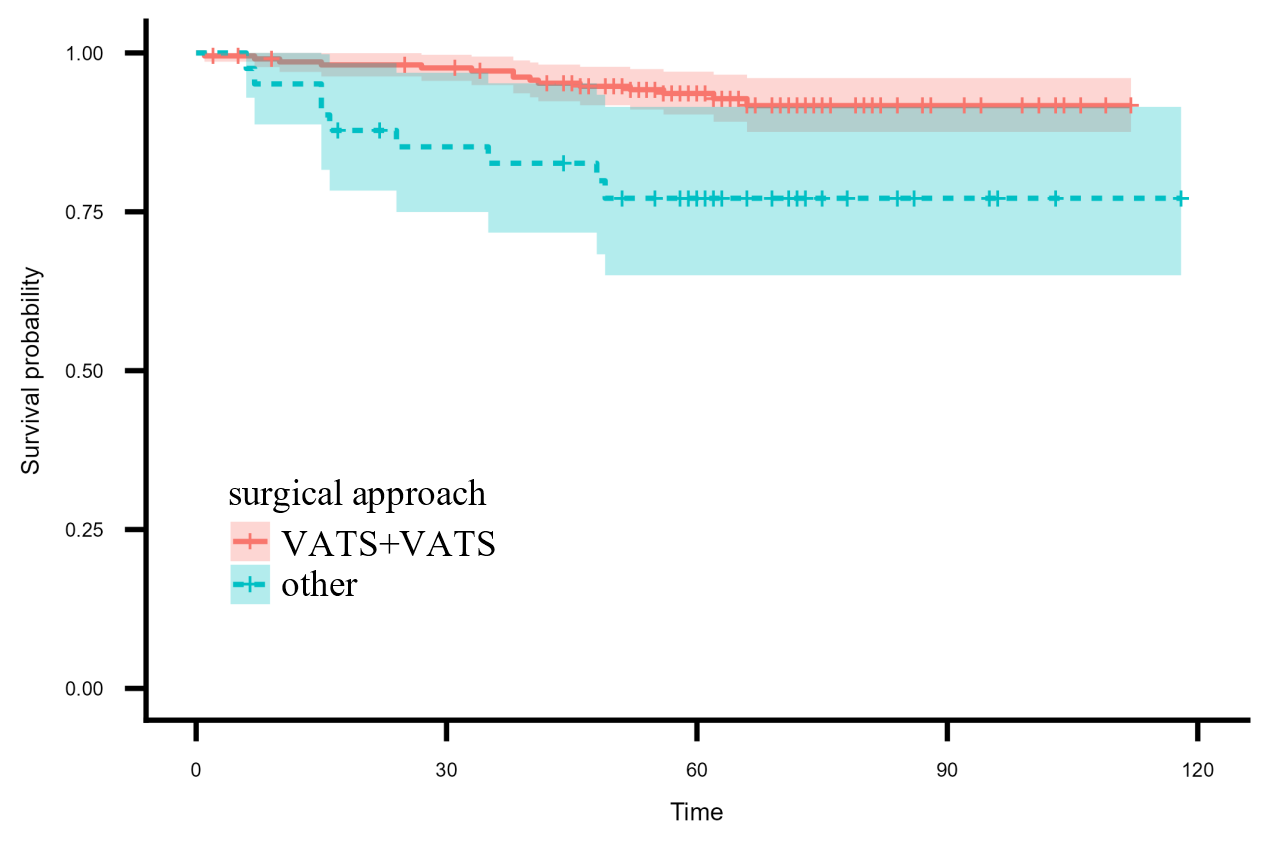


Figure S13. Kaplan-Meier analysis with log-rank test for the role of surgical approach with RFS (other vs VATS+VATS: HR: 3.481; 95%CI: 1.522-7.961; P=0.003). The horizontal axis is in months, and the vertical axis is in percentage.


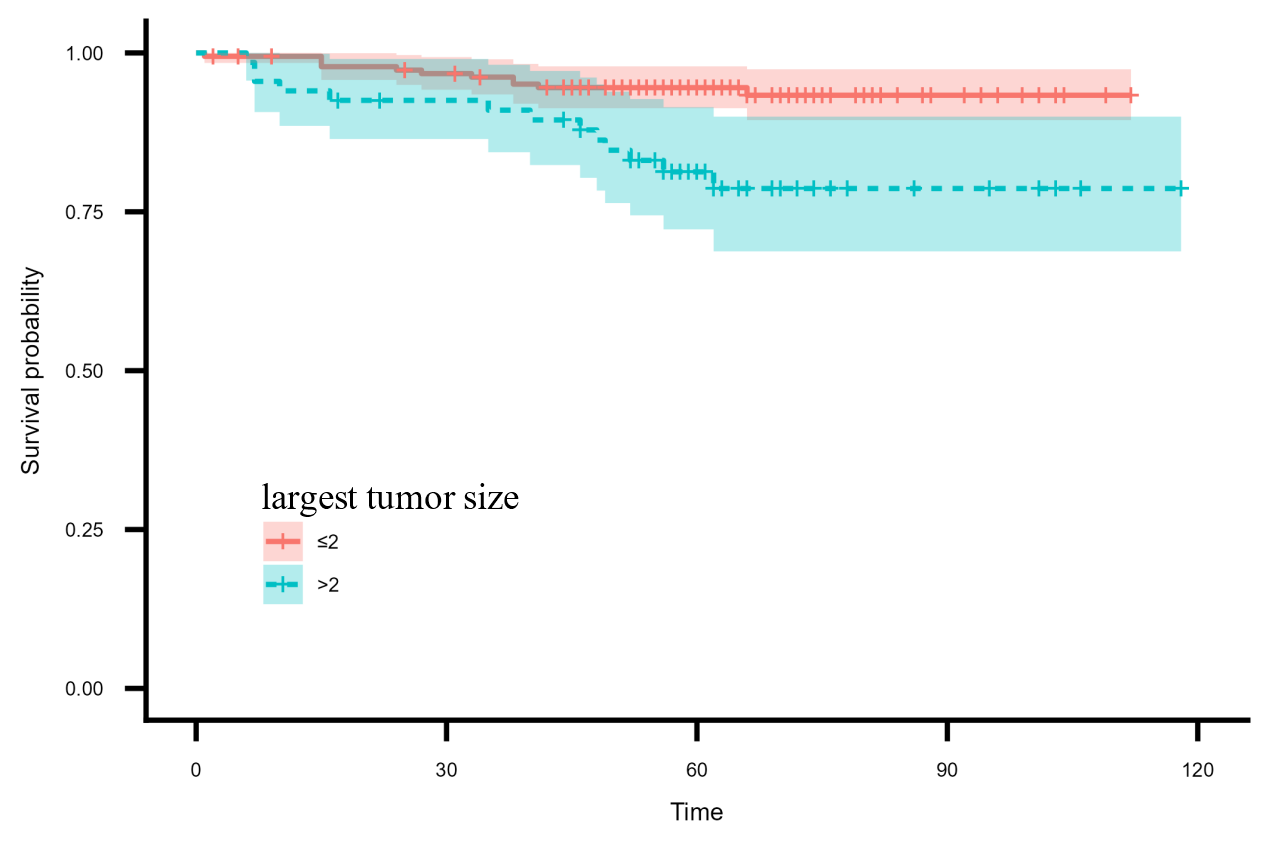


≤2

>2

largest tumor size

Figure S14. Kaplan-Meier analysis with log-rank test for the role of largest tumor size with RFS (>2 vs ≤2: HR: 3.469; 95%CI: 1.554-7.744; P=0.002). The horizontal axis is in months, and the vertical axis is in percentage.


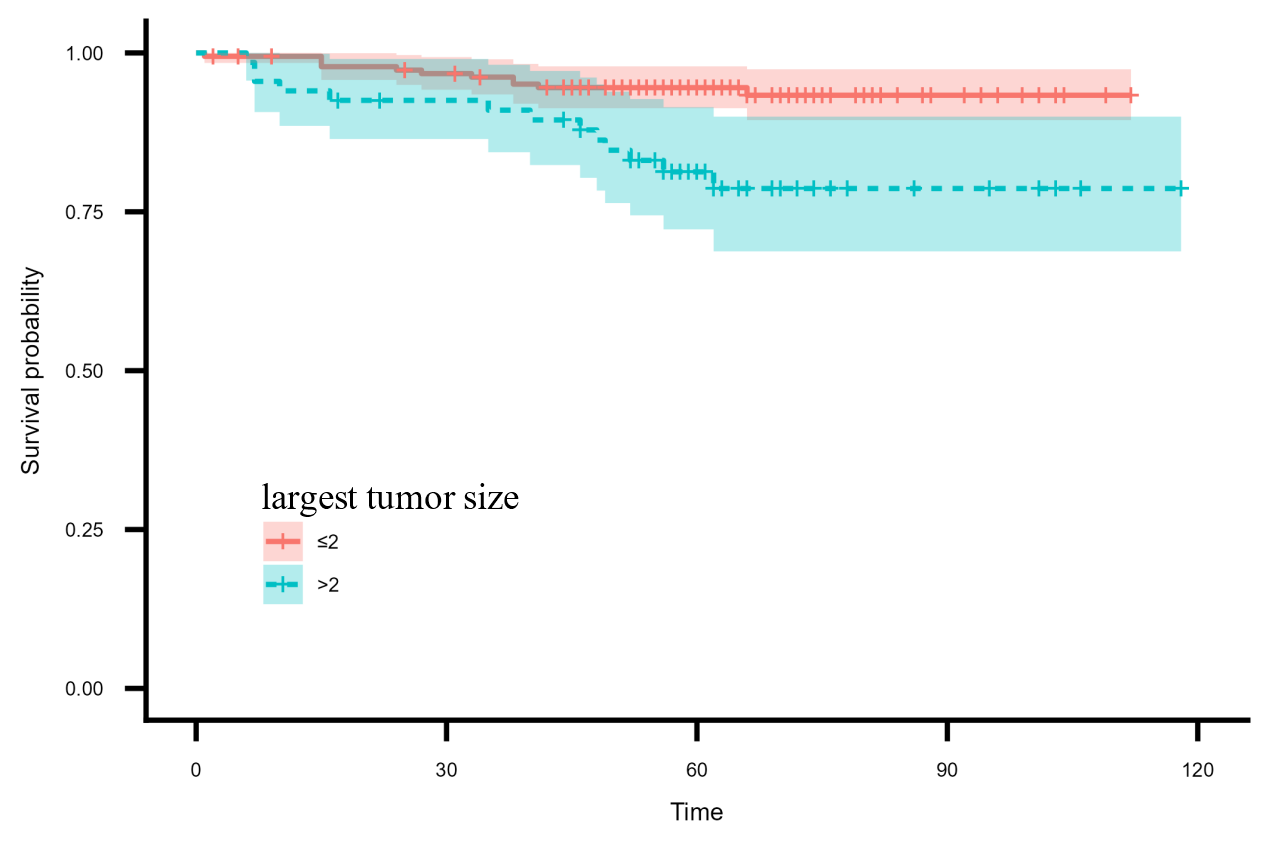


≤2

>2

largest tumor size

Figure S15. Kaplan-Meier analysis with log-rank test for the role of most advanced TNM stage with RFS (II+III vs I: HR: 5.964; 95%CI: 2.669-13.327; P<0.001). The horizontal axis is in months, and the vertical axis is in percentage.


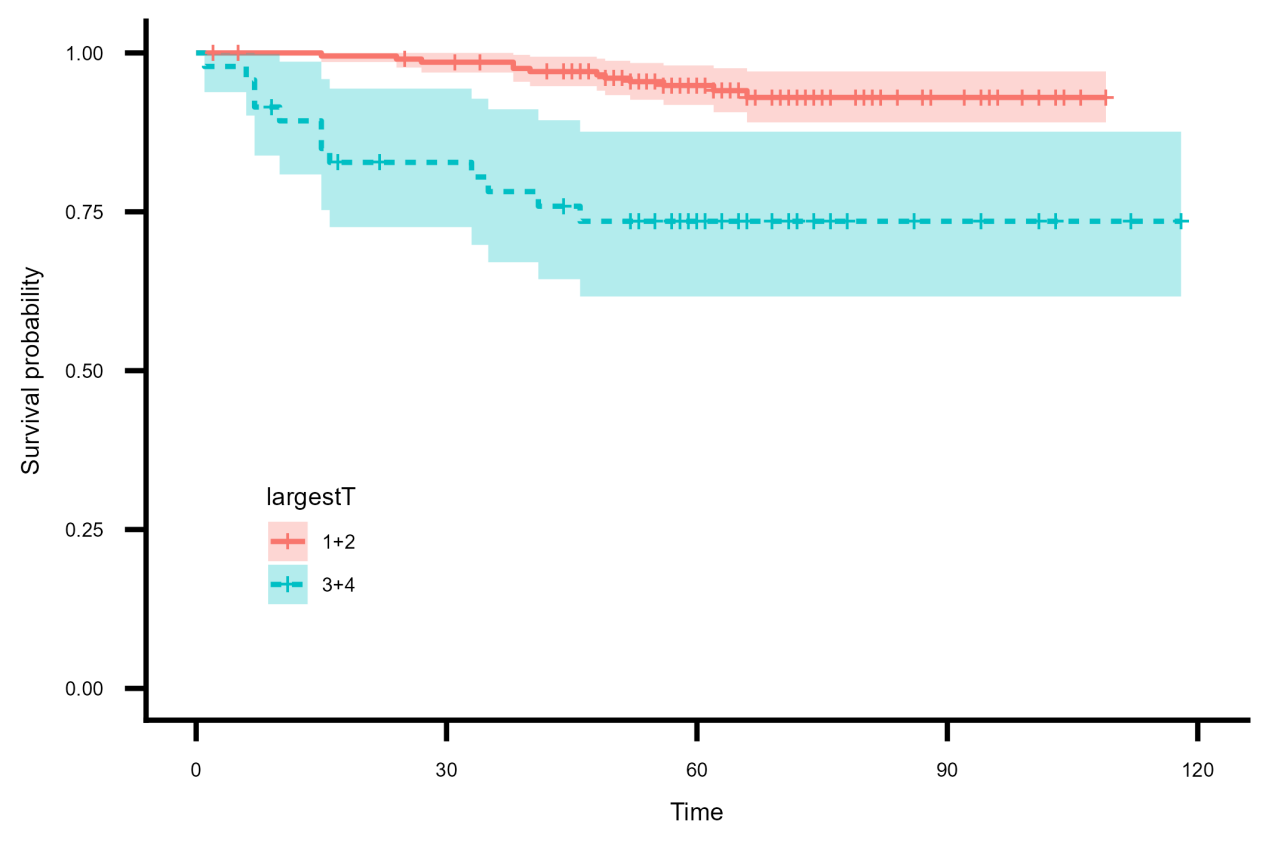


1+2

3+4

largestT

Figure S16. Kaplan-Meier analysis with log-rank test for the role of largest T stage with RFS (3+4 vs 1+2: HR: 5.460; 95%CI: 2.450-12.168; P<0.001). The horizontal axis is in months, and the vertical axis is in percentage.


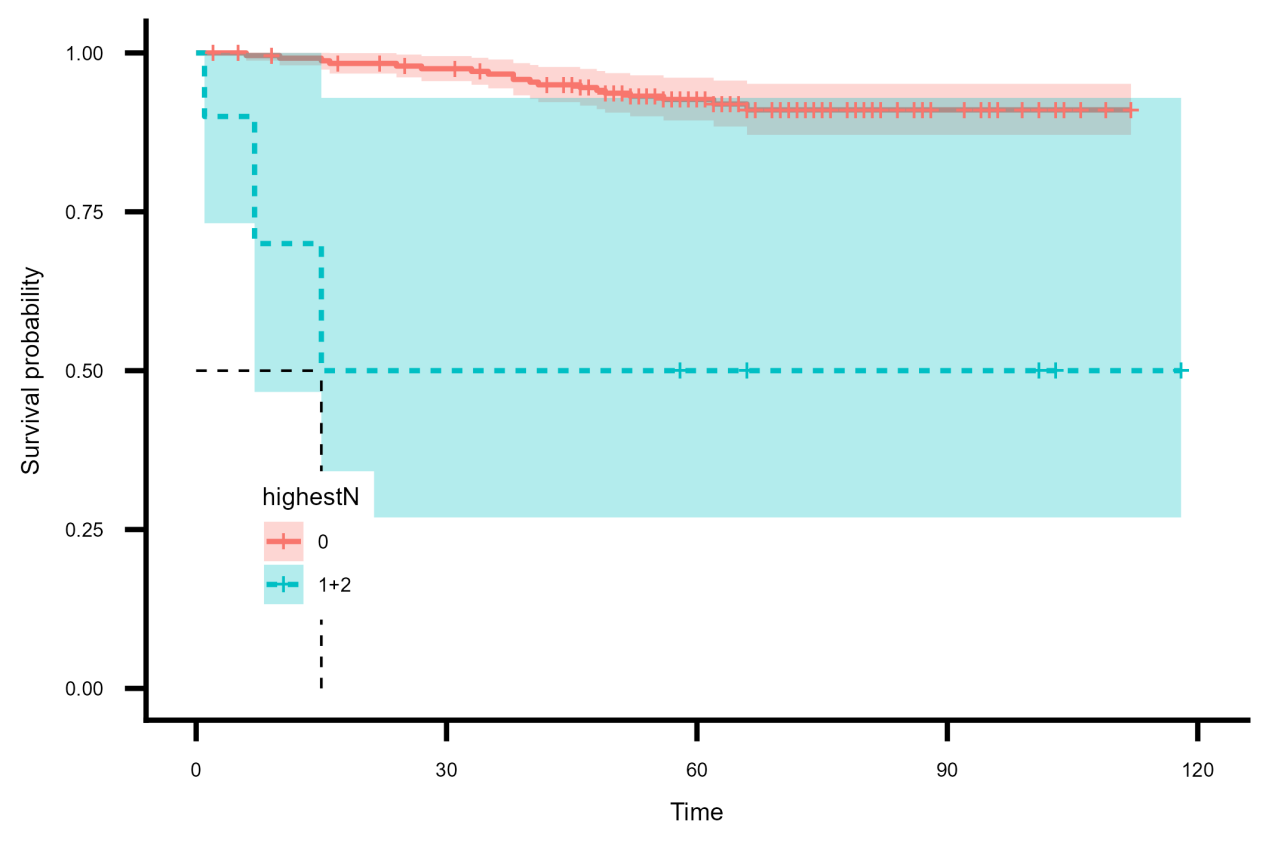


0

1+2

highestN

Figure S17. Kaplan-Meier analysis with log-rank test for the role of highest N stage with RFS (1+2 vs 0: HR: 9.726; 95%CI: 3.616-26.158; P<0.001). The horizontal axis is in months, and the vertical axis is in percentage.
